# Three-dimensional forward modeling and quantitative assessment of electrode offset effects in ERT

**DOI:** 10.1016/j.heliyon.2024.e35709

**Published:** 2024-08-03

**Authors:** Kui Suo, Mingdong Zhao, Menghan Jia, Wenhui Liu, Shizhong Chen, Guizhang Zhao

**Affiliations:** North China University of Water Resources and Electric Power, No. 136, Jinshui East Road, Jinshui District, Zhengzhou City, 450046, Henan Province, PR China

**Keywords:** Forward modeling, ERT, Electrode offsets, Topographic effects, Three-dimensional

## Abstract

Resistivity data has important applications in geophysical exploration, but the impact of electrode offsets on resistivity response characteristics remains unclear. This study aims to explore the influence of horizontal electrode offset angles and vertical offsets caused by topographical variations on the forward modeling of resistivity data. By analyzing experimental models with different measurement arrays, the paper revealed their influence laws on the buried depth of the target body and resistivity resolution. Utilizing tools like ZondRes3D, we conducted 3D resistivity forward modeling and analyzed the results in detail. It is found that horizontal electrode offsets lead to pseudo-anomalies in the apparent resistivity response, which is related to the offset angles and the number of electrodes. Under different conditions, the horizontal electrode offsets exhibit a “gradient variation” pattern. In addition, topographical variations can also cause distortions and offsets in the apparent resistivity curves and the locations of the anomaly response. Specifically, the measuring lines near the edge of the target bodies are more susceptible to these effects. Based on the comprehensive experimental results, we have drawn several conclusions regarding the impact of electrode offsets and topographical variations, including the effects of offset angles on the pseudo-anomalies, the anomalous response laws under different topographic conditions, as well as anomalous situations under specific angles. These findings provide crucial insights for interpreting resistivity data in geophysical exploration and addressing practical engineering problems, and offer guidance for optimizing measuring line layouts and post-processing terrain correction algorithms.

## 1 Introduction

In the current context of rapidly growing needs for monitoring slope stability, safety of large-scale engineering projects, and environmental restoration, the electrical resistivity method has been widely applied, with increasing demands for higher detection accuracy. Obstacles and topographical variations, however, may lead to horizontal and vertical offsets of electrodes, resulting in anomalies in the observed data. As these anomalies are becoming increasingly important, especially when the abnormal signals are weak, offsets can significantly impact the quality of resistivity measurement data, the correction becomes crucial. Quantitative evaluation of offset effects, therefore, has become a focus of current research. Forward modeling can help assess the impact of electrode offsets on resistivity data, laying the foundation for the development of correction algorithm research [1].Since Mufti et al. (1976, 1978) employed the finite difference method for two-dimensional forward numerical modeling of resistivity [2], Lu Jingjin (2009) and Song Tao (2015) and others successively utilized irregular grids to conduct three-dimensional resistivity forward modeling by the finite element method, analyzing and discussing the anisotropy effect [[3], [4], [5], [6]]. In addition, Yin Changchun (2016) and Liu Yajun (2019) et al. conducted forward modeling based on anisotropic models suited for stratified marine environments [[7], [8], [9], [10]]. Gerami Arshia (2018), and Kong Wenxin (2021) et al. developed and tested various algorithms, including equivalent resistivity network discretization, discretization based on finite element, and the finite element method, for three-dimensional DC resistivity and electromagnetic simulations in isotropic or anisotropic media, to characterize complex geological structures [[11], [12], [13], [14], [15]]. Chen Hui et al. (2017) introduced an aggregation-based algebraic multigrid method (AGMG), enhancing computational efficiency in three-dimensional DC resistivity simulations [16]. Shahriari et al. (2020) utilized the deep neural network to approximate the forward function generated by Maxwell's equations, improving the forward modeling efficiency of borehole resistivity measurements [17]. Varfinezhad and Oskooi (2020) developed a two-dimensional DC resistivity forward modeling code based on integral equations, and provided acceptable simulation accuracy through linearization, thus proving the practical feasibility of this approach [18]. Wang et al. (2020) investigated the impact of thermal effects on storage, advancing hardware development [19]. Mendoza et al. (2021) analyzed the strengths and weaknesses of different methods used to identify specific geological targets (such as mineralized structures) through the combined applications of resistivity, electromagnetic and seismic methods, providing multiple constraints for exploration [20]. Juliani, C. and Juliani, E. (2021) proposed a deep-sea terrain analysis method based on deep learning for deep-sea mineral exploration and utilized computer vision algorithms such as semantic segmentation and morphological similarity analysis to predict mineralization zones of submarine hydrothermal minerals, exploring a new approach to mineral exploration with artificial intelligence techniques [21]. Yang Yanfang (2022) studied the forward modeling response characteristics of different arrays' 3D ERT on filled cavities in various faults [22]. Tong and Sun (2024) employed a 2.5D DC resistivity forward algorithm combining finite difference approximation and the virtual point technology to numerically simulate two-dimensional models, providing qualitative interpretations for field data [23].With the advancement of these techniques, previous studies have made progress in the understanding of topographical effects, summarizing common scenarios: pure topographical anomalies occurs at locations of topographical changes, ridges has a greater impact on anomalies than valleys, and anomalies are greater in vertical terrain alignment layouts compared to parallel alignment layouts. Moreover, topographical anomalies are smaller in the middle of slopes and larger at mountain peaks and valley bottoms. Topographic anomalies are smaller in the centre of the slope and larger on the summit and valley floor.

Evidently, while numerical model techniques well-developed are in two dimensions, three-dimensional models are gradually becoming the mainstream, with comprehensive summaries of general rules for simple topographical conditions. Unfortunately, the quantitative evaluation of the impact of electrode offsets on various arrays’ resolution response in three-dimensions has not been adequately studied.

Given this, this paper divides offset into electrode horizontal one and vertical one caused by terrain, quantitatively evaluating the impact of deviation angles on the resolution of different arrays. Through the study of multiple models, including level ground, slope, step, ridge, Canal terrain, and valley, the impact of different measuring arrays’ electrode offsets on the response characteristics of buried target bodies with different depths and resistivity at different locations are systematically explored in this paper. The results have important theoretical and practical guidance for topographic correction in irregular sites, underground apparent resistivity distribution characteristics, and measuring line layouts in practical work.

## 2 Methodology

In this paper, the three-dimensional forward models are achieved through the ZondRes3D software, which is a program designed for interpreting three-dimensional Electrical Resistivity Tomography (ERT), induced polarization, and mise-a-la-masse methods, featuring high-density measuring line arrangement and complex topographical simulation capabilities. The modeling adopted a hexahedral grid discretization.

### 2.1 ERT forward modeling

Forward modeling involves calculating the geophysical response from models with known physical properties and geometric shapes. The program employs a finite element method (FEM) to numerically solve the partial differential equation that describes the conduction of electric fields in anisotropic media. This is achieved by dividing the medium into a network of cells with different resistivity and using a linear basis function within each cell to approximate the potential. This approach converts the original continuous partial differential equation problem into a algebraic equation system, enabling the utilization of a numerical algorithm to obtain the numerical solution. The expression is shown in Eqs. (1)–(1):

$$N\left(x,z\right)=\frac{\left(e+\text{fx}+\text{gz}\right)}{2E}$$

(1-1) $$\frac{\partial }{\partial x}\left(\sigma \frac{\partial \varphi }{\partial x}\right)+\frac{\partial }{\partial y}\left(\partial \frac{\partial \varphi }{\partial y}\right)-\frac{\partial }{\partial z}\left(\sigma \frac{\partial \varphi }{\partial z}\right)=-I\delta \left(x\right)\delta \left(z\right)$$

$$\frac{\partial \varphi }{\partial n}+v.\varphi =0$$

Where e, f, and g － constant coefficients, x － horizontal spatial coordinate, z － vertical spatial coordinate, φ － potential value, I － current value, σ － electrical conductivity medium of the medium, δ － Dirac delta function.

The subsequent solution for a set of spatial frequencies and the application of the inverse Fourier transform to the obtained spectral potential values yields the unknown values of the point source potential at the grid nodes [2]. As shown in Eqs (1) and (2):

(1–2) $$U\left(x,y,z\right)=\frac{2}{\pi }\int_{0}^{\infty }\varphi \left(x,\lambda ,z\right)\cos\left(\lambda .y\right)d\lambda$$

### 2.2 Procedure validation

**Fig. 1:**
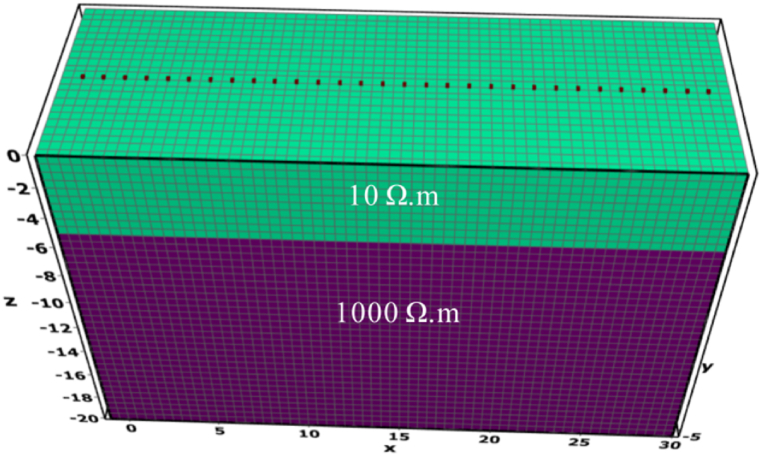
Validation model.

The validation model, as depicted in Fig. 1, is set within a range of 30 m × 5 m × 20 m, uniformly discretized with a grid size of 0.5 m × 0.5 m × 0.5 m. The model is divided into two layers, with the upper layer having a resistivity of 10 Ω m and a thickness of 5 m, while the lower layer a resistivity of 1000 Ω m and a thickness of 15 m.

Measurements were conducted through the Wenner-a array, with the point source located at coordinates (0, 1, 0). The measuring line was oriented along the X-direction, spanning 30 m, with electrode spacing set at 1 m.

**Fig. 2:**
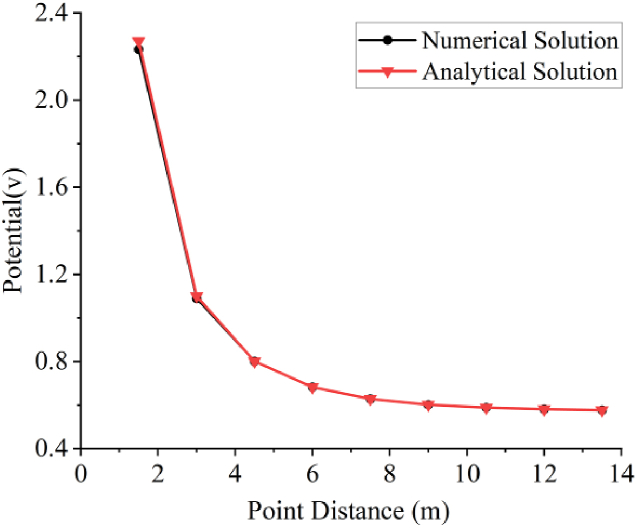
Comparison of numerical and analytical solutions for potential.

Potential values of different point distances were calculated through the finite element method forward modeling program presented in this paper and compared with the analytical solution. The results, as shown in Fig. 2, indicate an error of less than 0.05, primarily concentrated near the point source. The overall consistency of the results suggests the reliability of the data.

### 2.3 Modeling

Considering common observation errors encountered in practical work, this paper primarily focuses on the impact of horizontal and vertical electrode offsets.

**Fig. 3:**
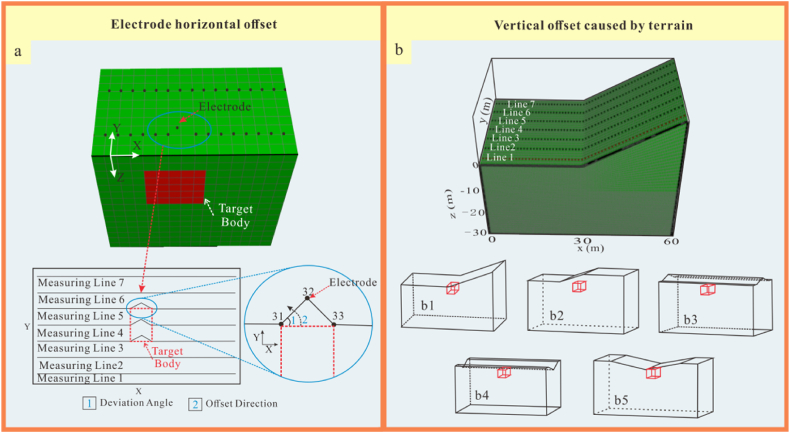
Schematic diagram of models.

For horizontal electrode offset, a horizontal model, as shown in Fig. 3a, was constructed to investigate four issues.

(1) The influence of the relative position between the measuring line and the target body.
(2) The effect of electrode deviation angle.
(3) The impact of the number of offset electrodes.
(4) The influence of different arrays.

Vertical electrode offsets are primarily caused by topographic variations. Thus, five models, as depicted in Fig. 3b, were constructed.

(1) Slope (Fig. 3b1): to evaluate the impact of different inclinations in the vertical direction on the response characteristics of the target body in comparison with level ground.
(2) Terrance (Fig. 3b2): to explore topographic response characteristics on the basis of adding topographic inflection points to the slope.
(3) Ridge (Fig. 3b3): to examine the resolution of measuring lines deployed along the ridge with different inclination angles.
(4) Canal terrain (Fig. 3b4): to evaluate anomalous response characteristics corresponding to the slope with different inclination angles in contrast to the ridge.
(5) Valley terrain (Fig. 3b5): to observe the anomalous response of measuring lines measured along different directions in comparison with the aforementioned terrains.

In the three-dimensional high-density resistivity forward modeling, various measurement arrays were employed. Among them, the Dipole-Dipole array exhibits high horizontal resolution and is sensitive to transverse resistivity changes induced by underground target bodies, making it suitable for detecting target bodies in vertical structures. Meanwhile, the Wenner array boasts high vertical resolution and a high signal-to-noise ratio, enabling precise measurements of target bodies in shallow layers [25]. Both arrays, therefore, were adopted in the model measurements.

Seven measuring lines were utilized for electrode placement, with the first electrode of the first line positioned at coordinates (0, 0, 0), followed by electrodes arranged sequentially. Each measuring line employed 60 electrodes with a spacing of 1 m, and the interval between measuring lines was set at 5 m.

The analysis of forward model includes the contour map of apparent resistivity and the introduction of the offset response parameter Ex, which provides a relative metric that eliminates the influence of absolute numerical values and enables comparisons of changes in different scales, thus making a clear understanding of the relative changes between two values possible. The offset response parameter Ex is calculated through the principle of percentage change (Eq. (2)):

(2) $$\text{Ex}=\left(\frac{\rho_{s}-\rho_{o}}{\rho_{o}}\right)\times 100\%$$

In Formula (2), Ex represents the offset impact parameter, $\rho_{s}$ indicates the apparent resistivity under the influence of offset at various angles, and $\rho_{o}$ refers to the apparent resistivity without offset. The physical meaning of the offset response parameter Ex is the contribution of the electric field distortion caused by the electrode offset to the apparent resistivity curve. Ex > 0 and Ex < 0 indicates an increase and decrease in apparent resistivity respectively, and Ex = 0 means no effect.

## 3 Results and discussion

### 3.1 Impact of electrode horizontal offset

**Fig. 4:**
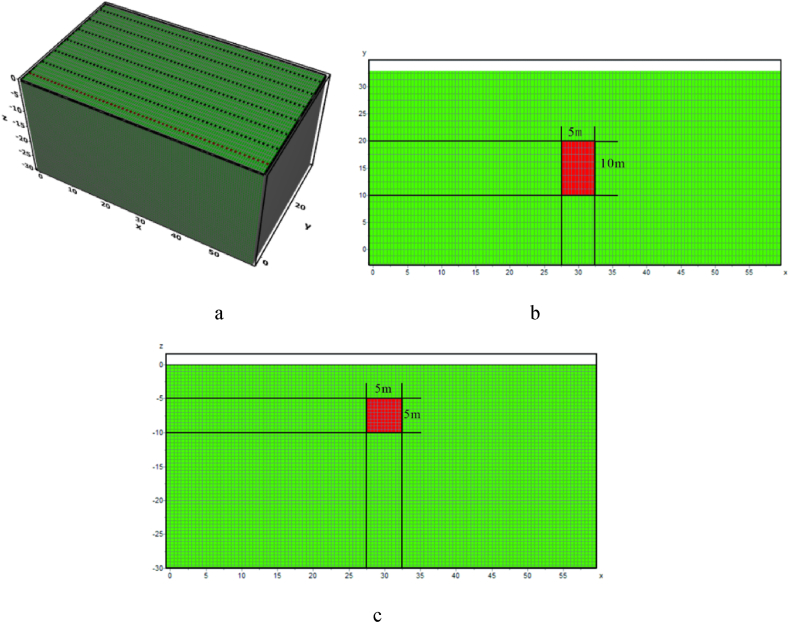
Horizontal model **a** Three-dimensional numerical model, **b** Model XY profile, **c** Model XZ profile.

Due to terrain uneveness, obstacles, and other uncontrollable factors, the horizontal offset of electrodes is a common phenomenon in practical measurements. To investigate the impact characteristics of electrode offset on ERT apparent resistivity responses, the following electrode offset schemes were designed based on the horizontal terrain model (Fig. 4):

The geo-electrical model dimensions were set at 60 m × 30 m × 30 m (Fig. 4a). The length of the target body was set to be 5 times the length of the minimum electrode spacing to ensure good resolution, with a volume of 5 m × 10 m × 5 m and a burial depth of 5 m (Fig. 4b、4c). The background resistivity of the model was set to 100 Ω m. Each electrode offset scheme involves measurements of the high and low resistances of the target body, with the high resistance set at 500 Ω m and the low resistance at 10 Ω m.

**Fig. 5:**
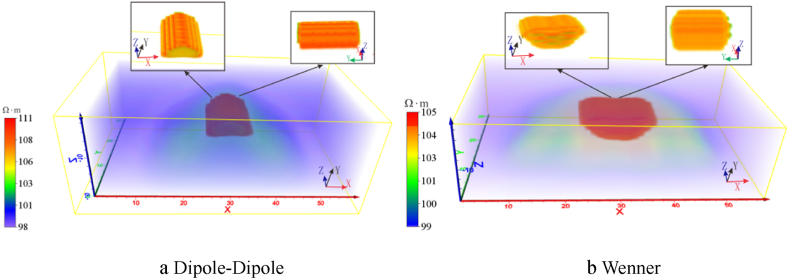
Three-dimensional apparent resistivity response of a high-resistivity target body under ideal conditions.

**Fig. 6:**
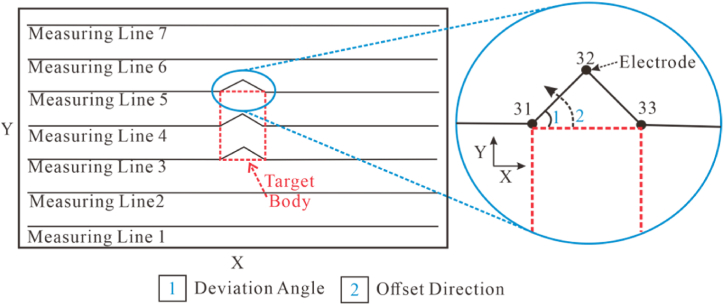
Schematic diagram of measuring line layout and electrode offset.

The electrode horizontal offset was measured through a variety of offset electrode counts, angles and positions from Dipole-Dipole and Wenner arrays. The measuring line layout is shown in Fig. 6. In the absence of offset, the response of the two arrays, as depicted in Fig. 5a and b, reveal that both failed to accurately reflect the shape of the target body. But they performed well in responding to the position and size of the target body, closely resembling the actual geological model.

#### 3.1.1 Impact of measuring line position

To investigate the response characteristics caused by the relative position of the offset electrodes and the ground projection of the target body, the following four schemes were designed.

(1) Offset electrodes were set on measuring lines 3, 4, and 5. Among them, lines 3 and 5 pass through the edges directly above the target body, while line 4 passes through the center. The specific layout is shown in Fig. 6.
(2) A piecewise interpolation method was adopted for the offset scheme. Initially, three datum angles (0°, 15°, 30°) were selected for forward calculation and its anomalous responses was observed. Interpolation was stopped when the anomalous results of consecutive interpolated angles showed no significant changes. Subsequently, eight angles with significant changes (0°, 12°, 15°, 21°, 28°, 30°, 35°, 45°) were analyzed.
(3) To increase the number of offset electrodes, scenarios where 1, 3, and 5 electrodes were offset were considered.
(4) The Dipole-Dipole array, with good horizontal resolution, was used to measure the above schemes. Then, the Wenner array was employed for the case of 5 offset electrodes to observe the response anomalies of different arrays.

**Fig. 7:**
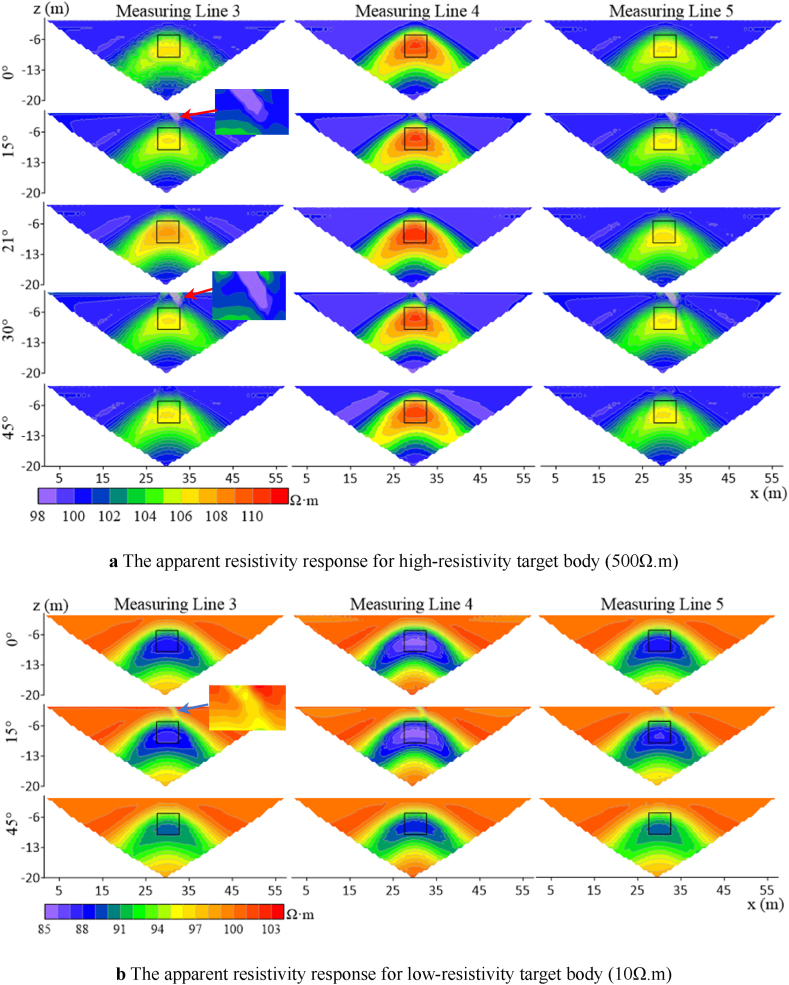
Characteristics of apparent resistivity responses of three measuring lines for high and low Resistivity target bodies.

According to Fig. 7a–as the angle increases, the impact on measuring line 4 is less than that on measuring lines 3 and 5 for the high-resistivity target body. The high-resistivity anomalies of lines 3 and 5 are shifted downward relative to the model position. Starting from 15°, the false anomaly of low-resistivity appears at the ground surface on the right side of the offset electrode for all three lines. As the deviation angle increases, this false anomaly disappears at a specific angle. At 21°, the response to the target body on line 3 is enhanced, with an increase in apparent resistivity and a restoration of the position response to match the model, while lines 4 and 5 show no significant changes. When the deviation angle reaches 30°, a false anomaly response of high-resistivity sandwiched between low-resistivity appears at the ground surface where the offset electrodes are located, and the apparent resistivity contours of line 4 begin to distort. At 45°, the false anomaly disappear, and the response of line 3 to the shape of the target body is significantly distorted, with significant distortions in the apparent resistivity contours.

When the target body resistivity is set to be low (as shown in Fig. 7b), the resistivity responses of lines 3 and 5 to the target body gradually weaken as the electrode deviation angle increases, while line 4 begins to weaken only after the angle reaches 45°. At 15°, a high-resistivity false anomaly appears at the ground surface where the offset electrodes are located. Unlike the high-resistivity target body, this phenomenon will not recur with the increase in the angle after 28°.

**Fig. 8:**
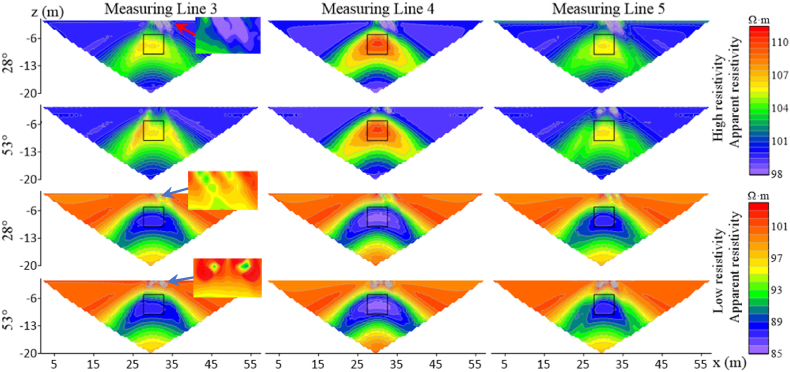
Characteristics of apparent resistivity response of three measuring lines for high and low resistivity target bodies with three electrode offsets.

When the number of offset electrodes is increased to three, the apparent resistivity response is shown in Fig. 8. The range of near-surface false anomalies of both high and low resistivity target bodies expands, and the distortion of measuring line 4 is the most severe here. The anomalous response of measuring line 3 for the high-resistivity target body shows a trend of stretching and breaking from the center as the angle increases, ultimately distorting into two high-resistivity anomalies at 53°. Measuring line 5 exhibits a weakening of the anomalous response, with the high-resistivity response shifting to the right. When the resistivity of the target body is low, the range of apparent resistivity anomalous responses expands as the electrode deviation angle increases. The density of apparent resistivity contours around the target body on line 3 shows a trend of being dense on the left and sparse on the right, while on line 5, the opposite trend is observed.

**Fig. 9:**
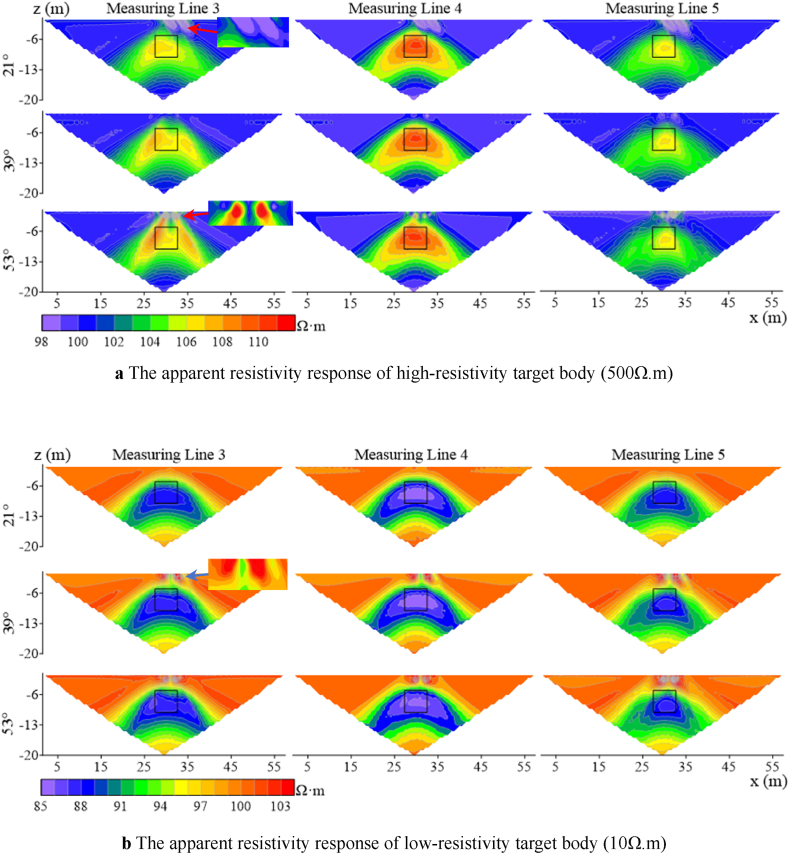
Characteristics of apparent resistivity response of high- and low-resistivity target bodies on three measuring lines at five electrode offsets.

When the number of electrode offsets increases to five and the target resistivity is high (500 Ω m) (Fig. 9a), it can be found that the anomalous responses of measuring lines 3 and 5 are distorted vertically, with the same trend as when the number of offset electrodes is 1 and 3 but to a more drastic extent. Fig. 9b shows that the distortion degree of the target body with low-resistivity is less than that of the target body with high-resistivity, and the distortion degree on measuring line 4 is less than that on lines 3 and 5.

**Fig. 10:**
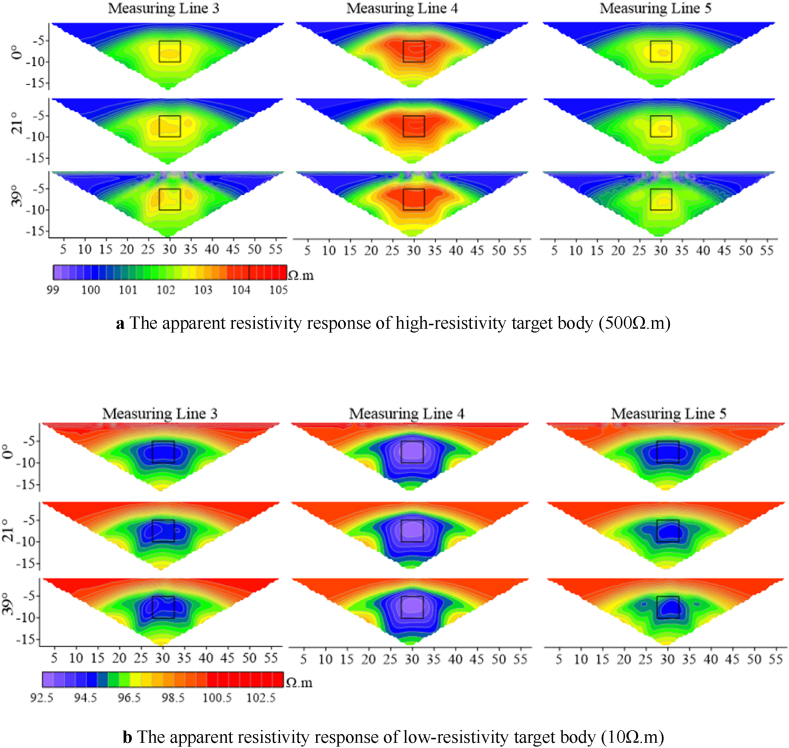
Characteristics of apparent resistivity response of high- and low-resistivity target bodies on three measuring lines at five electrode offsets under Wenner array's measurements.

As observed in Fig. 10, Wenner array's apparent resistivity contours are relatively smooth without jagged fluctuations. According to measuring line 3 in Fig. 10a, two high-resistivity anomalies appear at the target body location when the deviation angle is 21°, and the degree of distortion increases with the increase in the deivation angle. The shape of measuring line 5 exhibits irregular distortions as the deviation angle increases. Measuring line 4 has a large anomalous area, and its shape begins to distort as the deviation angle increases to 39°. When the resistivity of the target body is low(Fig. 10b), the response of line 4 does not change with the divation angle, indicating a high degree of recognition for the target body's location and size. As the deviation angle increases, lines 3 and 5 experience irregular distortions in their apparent resistivity contours, primarily manifesting as multiple low-resistivity anomalies horizontally and distorted edges.

Similarly, with five offset electrodes, false anomalies occur near the surface for Wenner array's high-resistivity target body at the deviation angle of 39°, while no false anomalies are observed for low-resistivity target body. In contrast, Dipole-Dipole array encounters false anomalies at 12°, indicating that Wenner array exhibits greater stability at shallow depths compared to the Dipole-Dipole array. When the target body resistivity is low, the response of Wenner array's measuring line 4 remains largely unchanged within the deviation angle of 40°compared to the non-offset case. The distortion extends primarily horizontally for Wenner array and vertically for Dipole-Dipole array.

In summary, measuring lines 3, 4, and 5 exhibit varying degrees of distortion in their electric fields, and both high- and low-resistivity target bodies show similar distortion trends as the deviation angle and number of electrodes increase. Line 4 is less affected than lines 3 and 5. The distortion of line 3 for high-resistivity target body is greater than for low-resistivity target body. As the angle and number increase, the apparent resistivity response at the target body location exhibits vertical depression and horizontal stretching. The distortion degree of response at the target body location on line 4 is lower for high-resistivity target body than for low-resistivity target body. The target body response on line 5 is offset towards the lower right corner.

These phenomena are primarily attributed to the distortion of the electric field caused by electrode offsets. Considering the relative positions of measuring lines 3, 4, and 5 to the ground projection of the target body, lines 3 and 5 are located on the two edges of the projection. As the electrodes offset, the offset electrode of line 3 move towards the center of the projection along the Y-axis, while the offset electrode of line 5 move away from the projection along the Y-axis. Line 4 passes through the center of the projection, and during offset, the electrode always cover the projected area of the target body on the ground.

#### 3.1.2 Impact of measuring line deviation angles

**Fig. 11:**
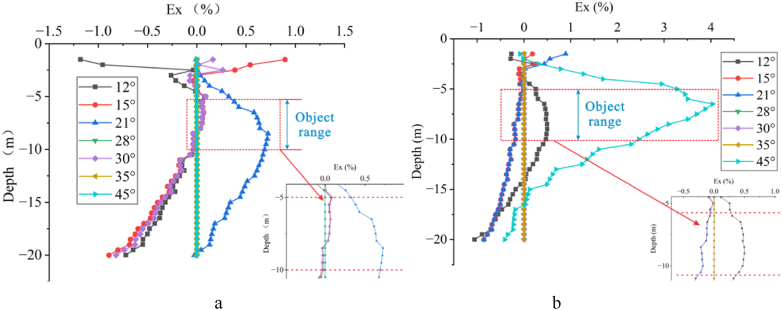
Ex curves for one offset electrode **a** High-resistivity target body, **b** Low-resistivity target body.

Based on the apparent resistivity response of measuring line 4 at different deviation angles, the Ex curves corresponding to each angle's depth axis at the offset electrode were calculated (Fig. 11). It can be observed that the Ex value fluctuates significantly near the surface. For high-resistivity target body (Fig. 11a), the angles corresponding to significant fluctuations are 12°, 15°, and 30°, while for low-resistivity target body (Fig. 11b), the angles are 12°, 15°, 21°, and 45°. It is evident that within 15°for high-resistivity and 21°for low-resistivity, the Ex value increases with increase in deviation angle, but beyond these angles, it decreases with further increase in deviation angle. Overall, the Ex value increases with the increasing angle, starts to decrease after reaching a certain angle, and then increases again with increase in angle, with some of the angles Ex = 0. As seen in Fig. 11, there are three distinct trends for high-resistivity and four trends for low-resistivity. At the location of the target body indicated by the red dashed line, the fluctuations are more significant for high-resistivity at the smaller angle (21°) and for low-resistivity at the larger angle (45°).

**Fig. 12:**
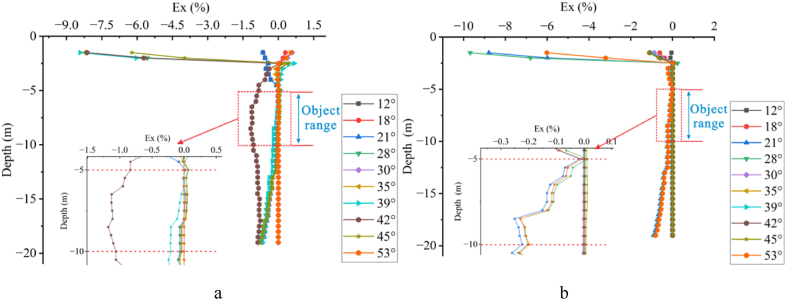
Ex curves for three offset electrodes **a** High-resistivity target body, **b** Low-resistivity target body.

As shown in Fig. 12, with three offset electrodes, there are varying degrees of fluctuations in the Ex value near the surface. For high-resistivity target body, significant fluctuations occur at 28°and above 39°, with the Ex value increasing with the angle, exhibiting a pattern of increase, decrease, and increase with boundaries at 18°, 35°, and 39°. For low-resistivity target body, significant fluctuations occur within the range of 21°–28°and at 53°, with all Ex values less than 0, showing a pattern of decrease, increase, and decrease with boundaries at 28°and 30°. Overall, there are three trends for high-resistivity (Fig. 12a) and two trends for low-resistivity (Fig. 12b), with significant fluctuations in the negative direction at the large angle (42°) for high-resistivity.

**Fig. 13:**
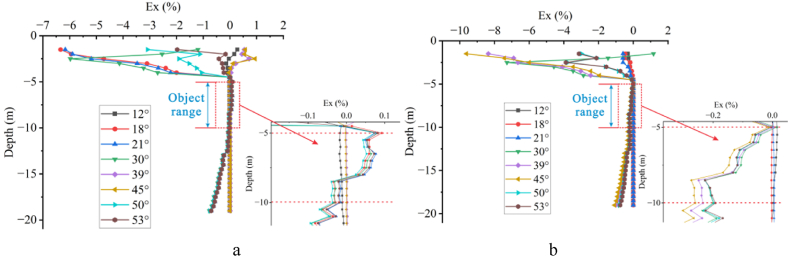
Ex curves for five offset electrodes **a** High-resistivity target body, **b** Low-resistivity target body.

As the number of offset electrodes increases to five, Fig. 13 reveals significant and complex fluctuations in the Ex value near the surface, accompanied by an increase in the depth of fluctuations. For high-resistivity(Fig. 13a), significant fluctuations occur between 18° and 39° and above 45°, with the Ex value increasing with the angle, exhibiting a pattern of decrease, increase, and decrease with boundaries at 18° and 45°. For low-resistivity(Fig. 13b), significant fluctuations occur above 21°, with the Ex value increasing with the angle, showing a pattern of decrease, increase, decrease, and increase with boundaries at 21°, 30°, and 45°. Overall, there are two main trends in the Ex curves for both high and low resistivity cases.

**Fig. 14:**
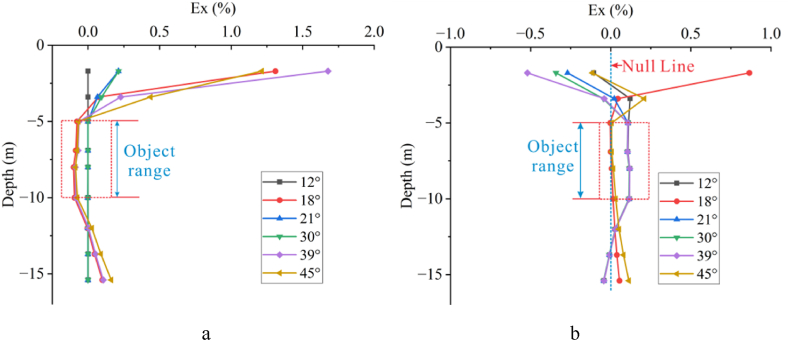
Ex curves for five offset electrodes of the Wenner array **a** High-resistivity target body, **b** Low-resistivity target body.

Shown in Fig. 14 are the Ex curves of the Wenner array measuring five electrodes offsets at six angles: 12°, 18°, 21°, 30°, 39°, and 45°. It can be observed that above the depth of −5 m, the Ex value of the high-resistivity target body(Fig. 14a) fluctuate in an M-shaped pattern with increase in the electrode deviation angle, remaining overall greater than 0 and decreasing with increasing depth. When the target body is of low resistivity(Fig. 14b), the Ex value exhibits an N-shaped fluctuation with increasing the deviation angle. The Ex value at 12°, 21°, 30°, and 39° increases with depth, all exceeding 0 at a depth of −5 m. But, when the depth exceeds −5 m, the Ex values for the high-resistivity target body at 18°, 39°, and 45° deviation angles are less than 0, indicating a weakened response. The Ex values for other deviation angles remain at 0, indicating no impact. But, the Ex values corresponding to 12°, 21°, 30°, and 39° deviation angles for the low-resistivity target body are greater than 0 within the depth range of −3 m to −13 m, suggesting a weakened response. Below a depth of −5 m, the two sets of Ex curves corresponding to each deviation angle for the low-resistivity target body exhibit consistent patterns, indicating a critical range for the impact of electrode offset on the response of the low-resistivity target body. Specifically, the degrees of impact are the same for 18° and 45° as well as for 12°, 21°, 30°, and 39°.

In summary, the Ex curves exhibit significant fluctuations and regular patterns in scenarios with single, three, and five offset electrodes. The Ex value of the high-resistivity target body exhibits larger fluctuations within a small angle range, initially increasing and then decreasing with the increasing angle. Conversely, the Ex value of the low-resistivity target body fluctuates significantly within a larger angle range. Not all deviation angles, however, affect the response. There are always deviation angles with Ex = 0 in Fig.s 11 to 14. In Fig. 11a, the deviation angles with Ex = 0 are 28°, 35°, and 45°; while in Fig. 11b, they are 28°, 30°, and 35°, with 28°and 35°overlapping, indicating that the two do not affect the target bodies with resistivity of 500 Ω m or 10 Ω m. In both Fig.s 11a and 11b, the deviation angle with Ex = 0 is 18°. But in Fig.s 13a and 13b, the overlapping deviation angle with Ex = 0 is 12°.

Electrode offsets alter the current path, resulting in particularly drastic changes in Ex near the surface. In apparent resistivity response, electrode offsets near the surface can lead to the false anomaly. As the deviation angle increases, the Ex value also increases, but when the angle reaches between 30° and 39°, the Ex value suddenly decreases and then increases again with further increases in the deviation angle.

#### 3.1.3 Impact of the number of offset electrodes

**Fig. 15:**
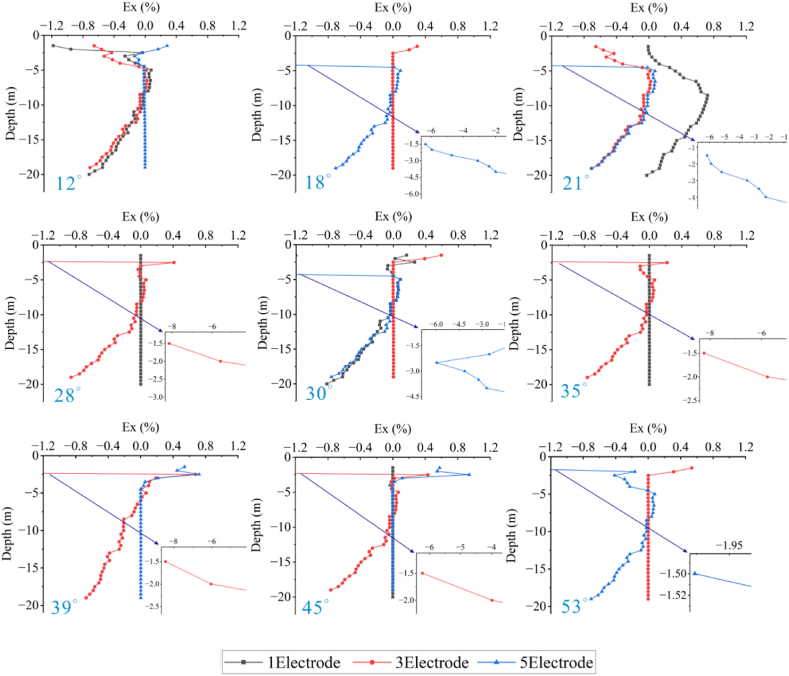
Ex curves for different numbers of offset electrodes at various angles for high-resistivity target body (Angles from left top to right bottom: 12°, 18°, 21°, 28°, 30°, 35°, 39°, 45°, 53°).

**Fig. 16:**
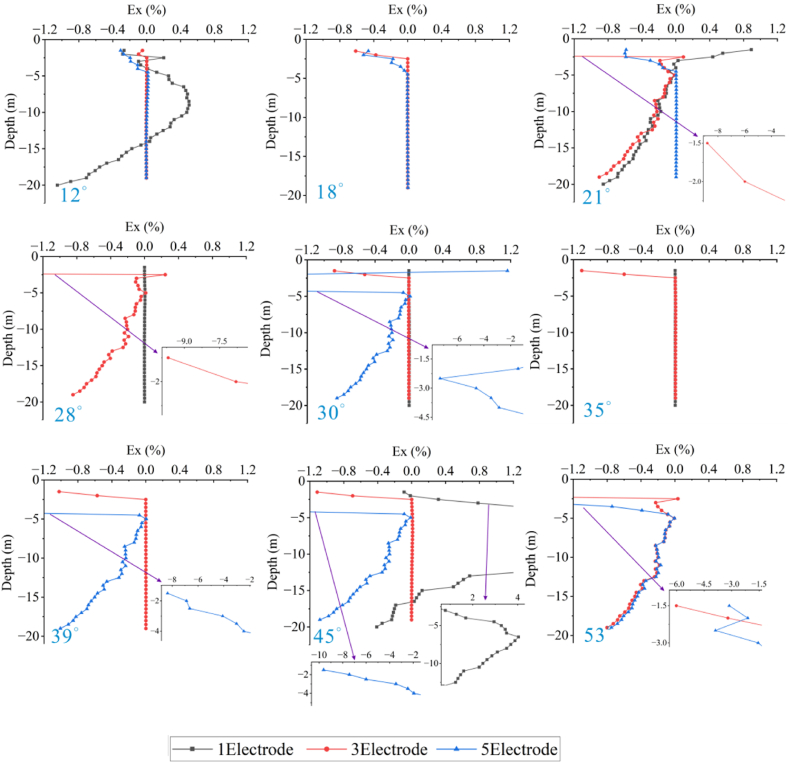
Ex curves for different numbers of offset electrodes at various angles for low-resistivity target body (Angles from left top to right bottom: 12°, 18°, 21°, 28°, 30°, 35°, 39°, 45°, 53°).

Keeping the deviation angle constant, the number of offset electrodes was increased. Three different numbers of offset electrodes, namely 1, 3, and 5, were used for each angle to investigate the response relationship between the number of offset electrodes and the deviation angle. As shown in Fig. 15, the impact of electrode offset is most pronounced near the surface. According to Fig. 16, when the deviation angle is less than 39°, the variation in Ex increases with the increase in the number of electrodes. But as the angle increases to 45°, the variation decreases with the increase in the number. As the angle continues to increase, the variation gradually increases with the increase in the number.

The curve where Ex = 0 indicates that the deviation angle has no effect on the apparent resistivity response. As can be seen from the Ex curves of different offset electrodes for high-resistivity target body in Fig. 15 and Table 1, when the deviation angle is less than 30°, the deviation angle where Ex = 0 decreases with the increasing number of offset electrodes, indicating that a greater number of offset electrodes are more likely to cause distortion. When the deviation angle is between 30° and 45°, the deviation angle corresponding to Ex = 0 first decreases and then increases with the increasing number. When the deviation angle is larger than 45°, the deviation angle first increases and then decreases to the same value as the deviation angle of a single offset electrode as the number of electrodes increases.

**Table 1:** Deviation angles corresponding to Ex = 0 for different numbers of offset electrodes for high-resistivity target body.

Angle	Angle1	Angle2	Angle3
Electrodeplate			
1	28°	35°	45°
3	18°	30°	53°
5	12°	39°	45°

When the target body has low resistivity (Fig. 16), the variation pattern of Ex near the surface is consistent with that of high-resistivity target body. As shown in Table 2, the deviation angles where Ex = 0 occurs for different numbers of offset electrodes are 28°, 12°, and 12° for the first occurrence, 30°, 18°, and 18° for the second occurrence, and 35°, 30°, and 21° for the third occurrence, respectively. It can be observed that as the number of offset electrodes increases, the deviation angle corresponding to Ex = 0 decreases, consistent with the pattern for high-resistivity target body. When the number of electrodes is 3, however, there are six deviation angles where Ex = 0, accounting for 2/3 of the total number of experimental groups, indicating a lower probability of distortion caused by electrode offset under this condition.

**Table 2:** Deviation angles corresponding to Ex = 0 for different numbers of offset electrodes for low-resistivity target body.

Angle	Angle1	Angle2	Angle3	Angle4	Angle5	Angle6
Electrodeplate						
1	28°	30°	35°			
3	12°	18°	30°	35°	39°	45°
5	12°	18°	21°			

A comprehensive analysis of the three different numbers of offset electrodes reveals that both high- and low-resistivity target bodies exhibit the same “gradient variation” pattern (curves where Ex ≠ 0 in Fig.s 15 and 16). Specifically, the variation in Ex near the surface is complex and intense, decreasing steadily after a inflection point at −5 m depth, exhibiting another inflection point at −8 m depth, and then a rapid decrease after a third inflection point at −10 m depth. The difference, however, lies in that for high-resistivity target body, Ex is greater than 0 between −5 m and −8 m, and suddenly becomes less than 0 at −8 m, while for low-resistivity target body, Ex is less than 0 after a depth of −5 m. Furthermore, as discussed in Section 3.1.2, with the increase in the number of electrodes, the trends of the Ex curves for both high-resistivity and low-resistivity cases become simpler and more unified, ultimately converging to only two trends: Ex = 0 and “gradient variation” when five electrodes are used.

#### 3.1.4 Impact of array types

**Fig. 17:**
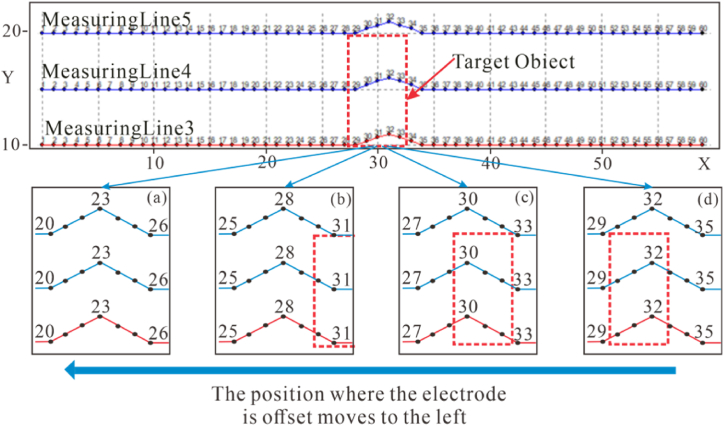
Schematic diagram of electrode offset positions The electrodes of inflection points **(a), (b), (c)** and **(d)** are 23, 28, 30 and 32 respectively.

This study employed both Dipole-Dipole and Wenner arrays for measurements. To observe the changes more clearly, five offset electrodes were selected, with other conditions remaining unchanged. The offset was induced by moving the electrodes to different positions (as shown in Fig. 17), aiming to investigate the characteristics of the target body's response affected by offset electrodes at different locations.

**Fig. 18:**
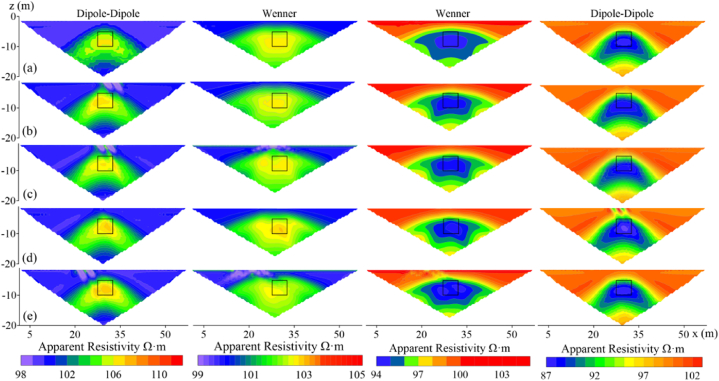
Apparent resistivity contour maps of offset electrodes at different positions on measuring line 3 **(a)** exhibits no offset but the inflection points of **(b), (c), (d)**, and **(e)** is at 32, 30, 28, and 23 respectively.

Fig. 18 presents the apparent resistivity contour maps of measuring line 3 for both high-resistivity (500 Ω m) and low-resistivity (10 Ω m) target bodies at the deviation angle of 21°, measured by the Dipole-Dipole and Wenner arrays respectively. Fig.s (a), (b), (c), (d), and (e) show the response when there is no offset, and the electrodes of inflection points are located at 32, 30, 28, and 23, respectively. It can be observed that compared to the case without electrode offset, the apparent resistivity contour maps at the four positions in all four scenarios exhibit varying degrees of distortion. Notably, the Dipole-Dipole array shows significant distortion in the vertical direction, while the Wenner array primarily manifests as horizontal distortion. As the offset electrode moves to the left, the location of false anomaly near the surface also shifts accordingly.

When the resistive of the target body is high, the most severe distortion occurs when the electrode of inflection point is located at 30 (X = 29 m, with an offset electrode range of X = 27m–31m). At this position, the target body's response is divided into two parts. As shown in Fig. 18, when the electrode of inflection point is positioned at 32 and 30, the number and manner of offset electrodes covering the target body are similar, with the only difference being that the target body is located to the left of the electrode of inflection point 32 and to the right of 30. One of the reasons for the difference is that the offset-induced current typically manifests as an extension along the measurement direction on the apparent resistivity contour map (as seen in the false anomalies near the surface in the Dipole-Dipole array high-resistivity target columns (b), (c), and (e) in Fig. 18). As the experiment is conducted from left to right, when the target body is located to the left of the electrode of inflection point, the false anomaly shape at offset location extends downward to the right, while when the target body is located below and to the left of the false anomaly, the anomaly does not directly extend to the target body's response position. When the target body is located to the right of the electrode of inflection point, however, the target body's response position coincides with the direction of anomaly extension, resulting in two high-resistivity anomaly responses.

When the resistive of the target body is low, the target body's response shape undergoes irregular distortion as the offset electrode position changes. At the electrode of inflection point 23, the Wenner array exhibits severe distortion, with two low-resistivity anomalies appearing at the target position. In contrast, the distortion degree of the Dipole-Dipole array remains relatively stable at various positions.

Based on the analysis, it can be found that different types of resistivity measurement arrays exhibit distinctly differential response characteristics when electrodes are offset. The Wenner array primarily exhibits horizontal distortion along the measuring line direction, which is related to its symmetrical electrode configuration and current conduction pattern. When encountering a high-resistivity target body, if the offset electrode is located on one side of the target body, current distortion may lead to the target response being split into two separate high-resistivity anomalies. For low-resistivity anomaly, its shape exhibits irregular distortion as the offset electrode shifts. The Dipole-Dipole array, however, due to its asymmetric electrode arrangement, tends to produce more significant measurement distortion in the vertical direction perpendicular to the measuring line. Regardless of whether the target is high- or low-resistivity, the distortion of its response is more reflected in the reduction of vertical resolution.

In practical work, therefore, when dealing with anomalous areas with offsets, it is necessary to conduct dense or even repeated measurements to obtain sufficient redundant data for cross-validation. If the expected target body is a high-resistivity anomaly, the Wenner array may be preferred; otherwise, the Dipole-Dipole array may be more suitable.

### 3.2 Impact of vertical electrode offsets

Terrain undulation alters the vertical position of electrodes, differing from horizontal electrode offsets. Topography-induced offsets are typically extensive and accompanied by changes in geological structures. Therefore, by comparing the influence characteristics of different topographies, we can observe their similarities and differences and complement each other to obtain precise results.

“Minimum Requirements for Geophysical Measurings” by Canadian Geophysical Union (CGU) emphasize that steep slopes greater than 15° require topographic corrections or changes in measuring line placement. However, current mainstream technical specifications do not provide sufficient theoretical basis or numerical simulation support. Therefore, it is necessary to systematically analyze the response characteristics and variation rules of topographic dip angles under different arrays.

#### 3.2.1 Impact of inclined terrain

The inclination model study includes four areas: (1) the effect of the dip angle on the results; (2) the effect of the same angle at different burial depths on the results; (3) the comparison of the results at different locations of anomalies at the same angle and burial depths; and (4) the comparison of the results between high-resistivity anomalies and low-resistivity anomalies under the same conditions. The parameter values of dip angle and burial depth in the model are set as shown in Table 3.

**Table 3:** Model parameters.

Angle	5°	10°	13°	15°	17°	20°	25°	30°	37°	40°
Burial Depth										
-2m	✓	✓	✓	✓	✓	✓	✓	✓	✓	✓
-3m		✓	✓							
-4m	✓	✓	✓							
-5m	✓	✓	✓							

**Fig. 19:**
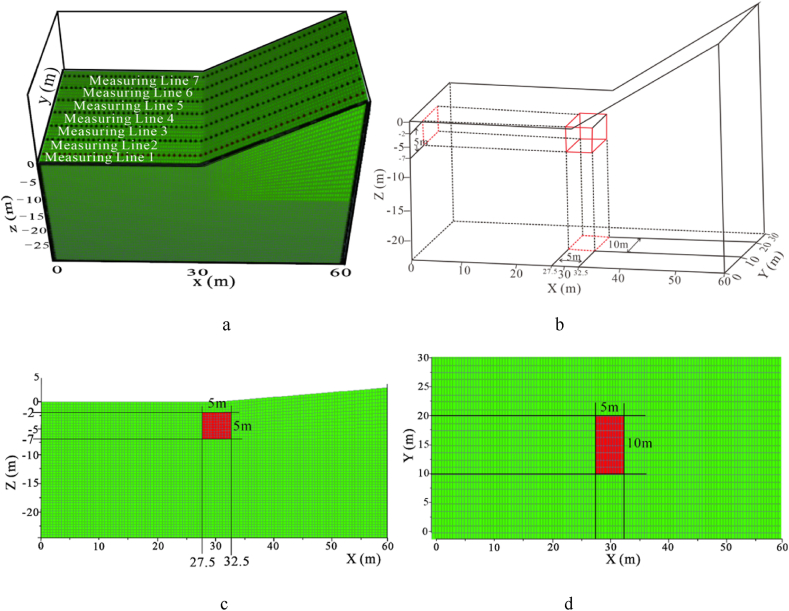
Schematic diagram of the location of the anomaly in the inclination model **a** inclined model, **b** anomaly 3D view, **c** model XZ profile, **d** model XY profile.

The anomaly is buried at a depth of 2 m and has dimensions of 5 m × 10 m × 5 m, as depicted in Figs. 19b, c,and 19d. A total of 7 measuring lines are covered, with 60 electrodes per line, an electrode spacing of 1 m, and a measuring line spacing of 5 m. Measurement methods include Dipole-Dipole and Wenner-a arrays, as detailed in Fig. 19a.

To observe the effect of topographic inclination on the results, the model was set with a burial depth of −2 m and a total of 10 sets of variations from low to high dip angles (5°–40°), as detailed in Table 3.

Forward models with different topographic dip angles were performed using the Dipole-Dipole array to extract the Ex curves corresponding to each dip angle at a depth of −4.5 m, as detailed in Fig. 20a, Fig. 20ba and 20b. It can be observed that the Ex values at the inflection points for all angles are less than 0. As the dip angle increases, the magnitude of the Ex curve distortion at this position also increases, and the horizontal variation range is consistent with the size of the target body. The Ex values on the left and right sides of the inflection point are greater than 0, and and the magnitude of curve distortion increases with angle.

For the high-resistivity target body (see Fig. 20a), the Ex values at the inflection point exhibit a pattern of increasing, decreasing, and then increasing, with 20° and 25° as the boundaries. The distortion amplitude on the left side of the inflection point is greater than that on the right side. The Ex values corresponding to each angle at the right edge are less than 0, and the distortion amplitude is opposite to the slope tendency and increases with the angle (see Fig. 21).

**Fig. 20a:**
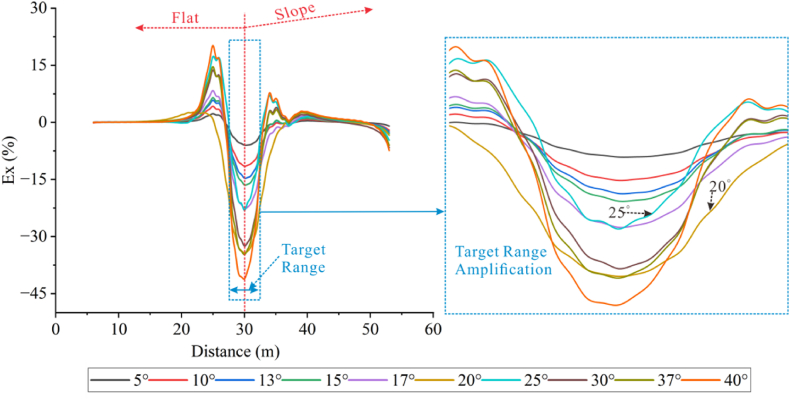
Variation curves of Ex for different dip angles of the high-resistivity target body in the Dipole-Dipole array.

**Fig. 20b:**
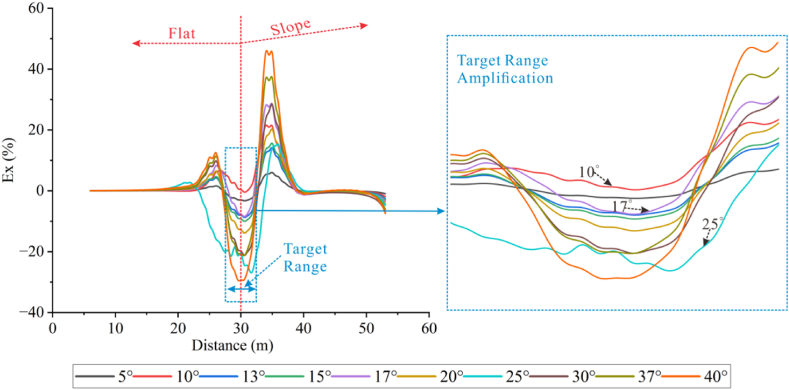
Variation curves of Ex for different dip angles of the low-resistivity target body in the Dipole-Dipole array.

For the low-resistivity target body (see Fig. 20b), the Ex values at the inflection point decreases and increases in a wavy undulation in steps of about 5° within 30°, and in steps of about 10° after 30°. The distortion amplitude on the left side of the inflection point is much smaller than that on the right side, and corresponds to Ex < 0 for each angle. The distortion amplitude at the right edge is opposite to the slope tendency and increases with the angle.

The results of the Wenner array are shown in Figs. 20c and 20d. The depth of the Ex curve is −4.5 m. It can be found that the Ex values corresponding to each angle at the inflection point are greater than 0. As the dip angle increases, the distortion amplitude of the Ex curve at this position also increases, and the horizontal variation range is consistent with the size of the target body. The Ex values on the left and right sides of the inflection point are less than 0, and the distortion amplitude of the curve also increases with the angle. There are abrupt changes in the curves at the edges on both sides of the target body.

For the high-resistivity target body (see Fig. 20c), the Ex values at the inflection point show a wavy rise and fall with a step of approximately 3°, opposite to the pattern observed for the low-resistivity target body in the Dipole-Dipole array. The step length becomes longer near 30°. The Ex values corresponding to each angle on both sides of the inflection point are less than 0, and the distortion amplitude is greater than that at the inflection point. The distortion amplitude on the left side is less than that on the right side, and the distortion amplitude at the right edge is the same as the slope tendency, while increasing with the increase of the angle.

**Fig. 20c:**
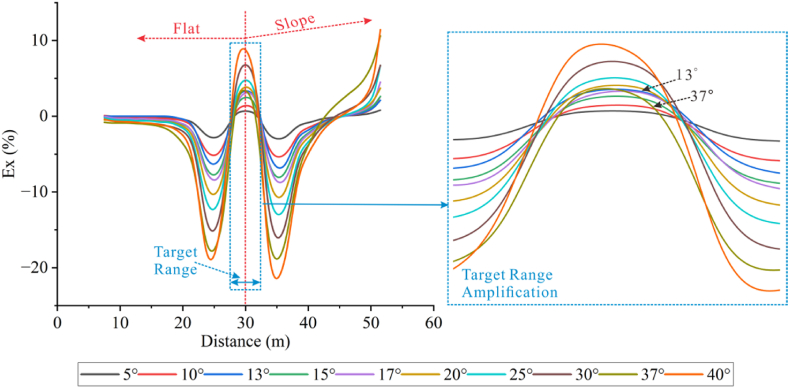
Variation curves of Ex for different dip angles of the high-resistivity target body in Wenner array.

For the low-resistivity target body (see Fig. 20d), the Ex values at the inflection point position show a wavy rise and fall with a step size ranging from approximately 3°–10°. Notably, except for 40°, the distortion amplitude of all angles on the right side of the inflection point is less than that on the left side. Additionally, at the right edge of x = 40m, the simulation results show a positive distortion curve with Ex > 0, and its amplitude not only aligns with the direction of the topographic inclination but also increases gradually with the increase of the angle.

**Fig. 20d:**
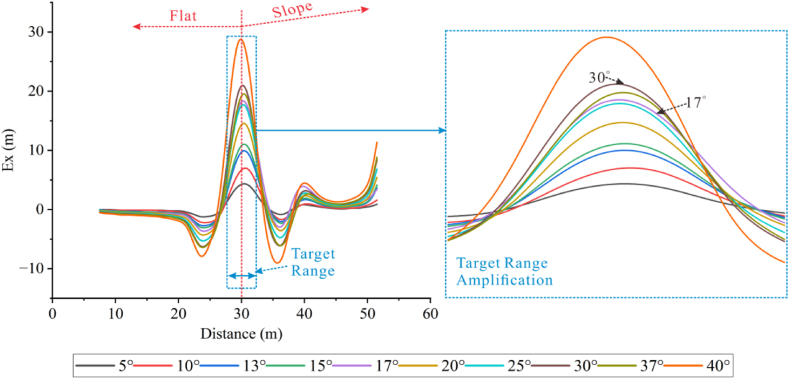
Variation curves of Ex for different dip angles of the low-resistivity target body in Wenner array.

Based on the Ex variation curves of the two arrays, it can be found that the locations of the distortion occurrence includes the inflection point, both sides of the inflection point, and the slope side. The shape of the curves and the trend of variation with inclination are similar for the same array for high and low resistivity targets, but the amplitudes are different. The distortion directions of the Ex curves of the Dipole-Dipole and Wenner arrays are opposite, which is caused by different rules for recording points. At the topographic inflection point, the AB and MN of the Dipole-Dipole array are located on both sides of the inflection point, while the MN of the Wenner array crosses the inflection point. The curve amplitude at the edge position on one side of the topographic slope increases with the direction of the slope inclination. The degree of Ex distortion on the right side of the inflection point indicates an enhancing trend for high-resistivity target bodies and a suppressing trend for low-resistivity target bodies, resulting in a slight increase in the apparent resistivity on one side of the topography.

Based on the apparent resistivity response results (see Figs. 21a, Figs. 21b), it can be observed that at 20°, the high-resistivity target body anomaly response of the Dipole-Dipole array is completely suppressed by the topography, and there is no high-resistivity anomaly response at the original position. At around the inflection point, the resistivity distribution horizontally presents a high-low-high pattern. The low-resistivity target anomaly location can be responded to with an apparent resistivity horizontal distribution of high-low-high. Under the influence of terrain, the high-resistivity anomaly response of the Wenner array is vertically distorted, and the low-resistivity anomaly is horizontally distorted, but the target body position can still be discerned. The apparent resistivity horizontal distribution of the high-resistivity target body presents a low-high-low pattern, while the low-resistivity target body presents a high-low-high pattern.

**Figs. 21a:**
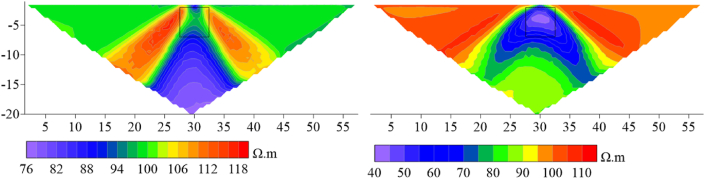
Contour plot of apparent resistivity of high-resistivity (left) and low-resistivity (right) targets at 20° inclination in the Dipole-Dipole array.

**Figs. 21b:**
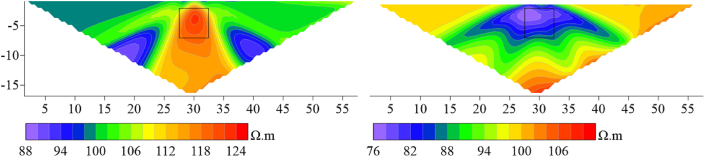
Contour plot of apparent resistivity of high-resistivity (left) and low-resistivity (right) targets at 20° inclination in the Wenner array.

To investigate the effect range of topographic factors, the slope dip angle was fixed, and the burial depth of the target body was increased to explore the vertical range. Meanwhile, with a fixed burial depth, the horizontal position of the target body is varied to study the lateral effect range.

Use the Wenner array. The Ex curves corresponding to different burial depths (see Figs. 22a) reveal that for the high-resistivity target body, the Ex values decrease as the burial depth increases in the range of −2 m (black line) to −5 m (green line), and become less than 0 after exceeding the depth of −3 m. When the burial depth reaches −6 m, the Ex amplitude suddenly increases to its maximum value and becomes greater than 0. Subsequently, as the burial depth increases, the Ex values gradually decrease. For the low-resistivity target body, the Ex distortion amplitude increases with increasing burial depth within the range of −2 m to −5 m, but when the burial depth is greater than −6 m, the distortion amplitude begins to decrease. Changing the burial depth of the target body results in changes in the Ex curves corresponding to different burial depths for both high and low resistivity. The peaks at the inflection points gradually slow down as the depth increases, eventually forming a double peak. This indicates that the depth of the effect at the inflection point is less than −6 m, because as the depth approaches −5 m, the Ex curves for both high and low resistivity target bodies gradually overlap with no further change in amplitude until a sudden change occurs at a depth of −6 m.

**Figs. 22a:**
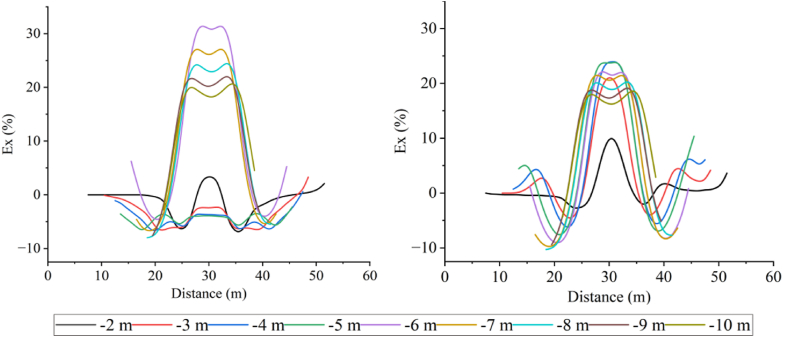
Ex curves of high-resistivity **(left)** and low-resistivity **(right)** target bodies with different burial depths at 13° inclination in the Wenner array.

Fix the burial depth to −2 m and change the burial position of the target body horizontally. The Ex curves at different horizontal positions of the target body were extracted, as shown in Figs. 22b. The curve crests appear at the target body position, the inflection point, and both sides of the inflection point.

**Figs. 22b:**
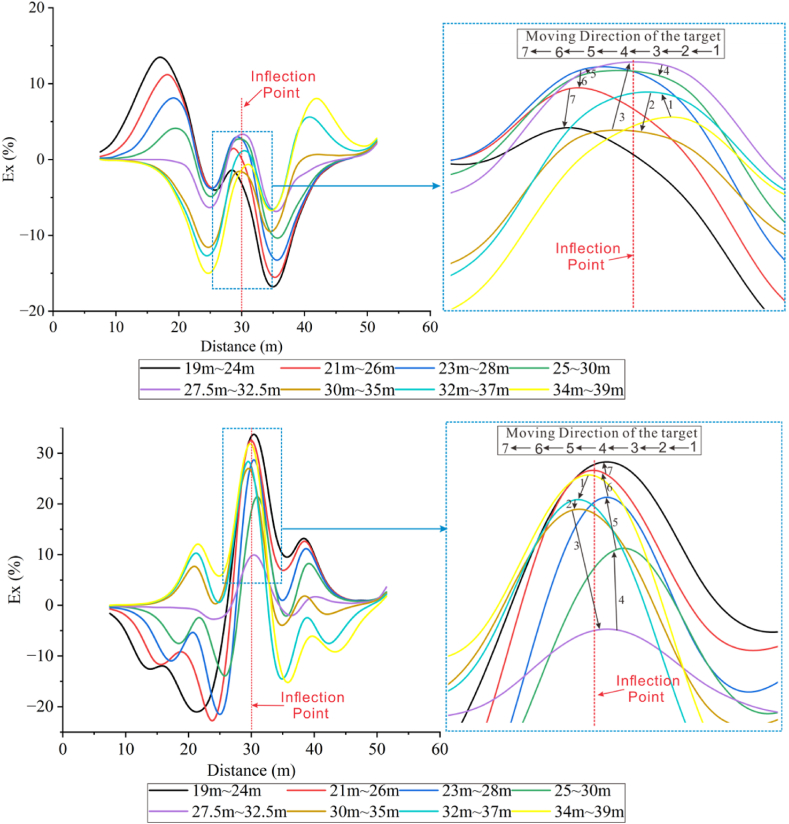
Ex curves for different horizontal positions of high-resistivity **(up)** and low-resistivity **(down)** target bodies with 13° inclination in the Wenner array.

As the position of the target body moves to the left, the positive main peaks of the curves corresponding to the different positions of the high-resistivity target body (see Figs. 22a (top)) move to the left accordingly, and the closer it is to the inflection point, the smaller the peak value becomes. The peak on the topographic side is smaller than that on the flat terrain side. This indicates that changes in topography alter the propagation path of the current in the underground, affecting the distribution of current density. At the topographic inflection point, the current density is high, and it is low on the slope side, while the density on the slope is less than that on the flat terrain. Reflected in the Ex curves, the high current density corresponds to the high curve amplitude, which has a suppressing effect on high-resistivity target bodies. The curves between 19 m and 34 m on the X-axis all vary with the target body position, indicating that the horizontal influence range is at least 8.5 times the electrode spacing on one side of the inflection point. At the inflection point, the relative position of the peaks also remains consistent with the direction of target body movement, and the peak amplitude increases as it approaches the inflection point, but the amplitude of the wave peaks in the two positions closest to the inflection point, 25 m–30 m and 30 m–35 m, suddenly decreases (high resistivity right Fig. 3, Fig. 5 process). This is because the current density increases at the positive topographic inflection point, and the high-resistivity body repels the current, resulting in a decrease in the current density around the high-resistivity body as it approaches the inflection point.

For the changes in the corresponding Ex curves (see Figs. 22b (bottom)) for each position of the low-resistivity target, the remaining curves except for 27.5 m–32.5 m (purple line) were mainly observed with the trough of Ex < 0. It can be noticed that the position of the trough moves in the same direction as the target body moves left. The amplitude of the trough is positively correlated with the distance from the inflection point, and the closer the distance is, the shallower the trough is, and the trough on the terrain side is shallower than that on the flat ground side. This is due to the presence of the inflection point attracts current, the closer the distance the greater the effect, while the low-resistivity target body Ex is negative and the current density around the target body increases as the distance gets closer, which reacts as a shallower trough on the curve.

At the inflection point, the Ex values are greater than 0. The target body directly below the inflection point corresponds to the smallest peak in the Ex curve, and the peak increases as the distance from the inflection point decreases. This phenomenon is due to the fact that the low-resistivity target attracts current, which reduces the current density around the target. The closer the distance is, the smaller the current density at the inflection point becomes, resulting in a smaller peak.

Through comprehensive analysis of the forward data, electrode placement should span the inflection points for exploration work near topographic inflection points.

#### 3.2.2 Impact of step topography

Based on the one-way sloping model, a portion of the sloping surface was modified into flat terrain, with the anomalies essentially unchanged (see Figs. 23a). The slope ranged from 20 m to 35 m on the X-axis, with dip angles varying from 5°, 10°, 13°, 15°, 17°, 20°, 25°, 30°, 35°, and 40°. The target body resistivity was set at 500 Ω m for high resistivity and 10 Ω m for low resistivity, while the background resistivity was 100 Ω m.

**Figs. 23a:**
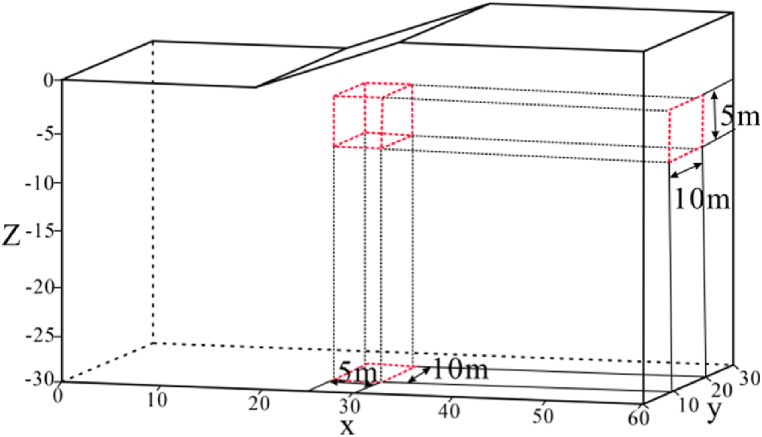
Schematic diagram of the structure of the inverse Z-shaped model.

This model used a more stable Wenner array for vertical topographic measurements. By fixing the position of the anomalies and varying the dip angles of the slope, the apparent resistivity response characteristics of different angles were obtained, and the results with significant changes were selected for presentation.

According to Figs. 23b (left), it can be observed that the high-resistivity anomalous response at the target body position shifts to the right, with irregular shape distortion and multiple high-resistivity anomalies appearing as the angle increases. When the angle reaches 40°, the target body is completely shielded by the topography. At the inflection point where the low flat area connects to the slope (hereinafter referred to as the left inflection point), the apparent resistivity increases with the increase of the angle, and the resistivity extends from the bottom to the surface, with decreased apparent resistivity on both sides of the inflection point. At the inflection point where the top of the slope connects to the high flat area (hereinafter referred to as the right inflection point), the resistivity decreases as the angle increases, and it extends from the surface downward, with increased resistivity on both sides of the inflection point. Under the influence of topography, the resistivity exhibits a distribution pattern of low-high-low-high-low-high.

**Figs. 23b:**
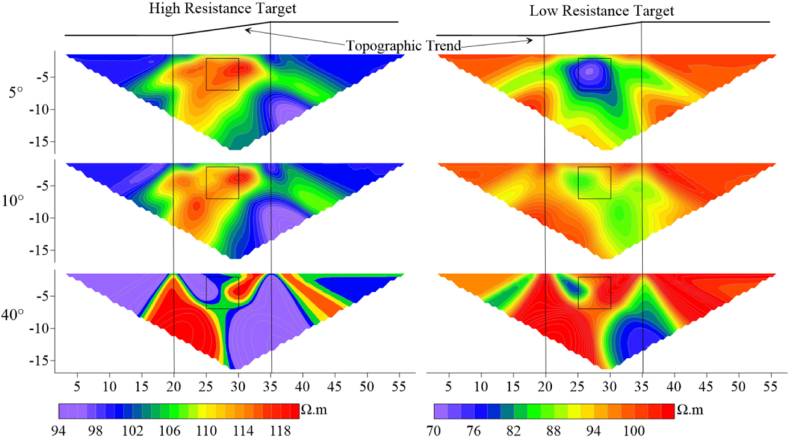
Results of the apparent resistivity response at different angles for high-resistivity **(left)** and low-resistivity **(right)** targets of the measuring line 4.

Observations show that the response at a tilt angle of 5° is better than that of the high resistivity when the target body is low resistivity (Figs. 23b (right)). The position and size of the low-resistivity anomaly are basically the same as those set in the model. As the angle increases, the response of the target body gradually disappears. The variation pattern of apparent resistivity is simiar to that of high resistivity.

**Fig. 24:**
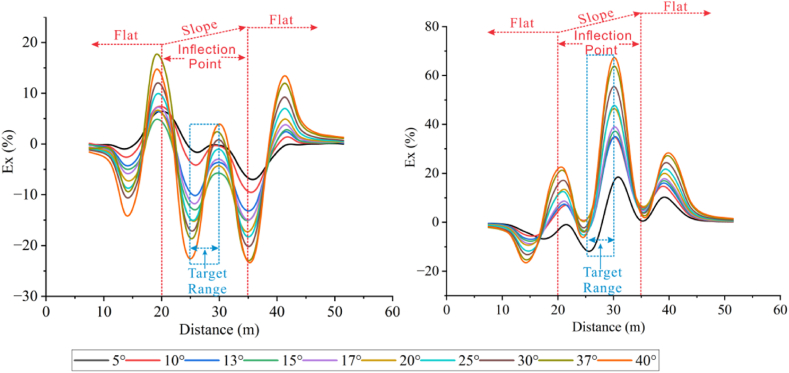
Ex curves for high-resistivity (left) and low-resistivity (right) target bodies at different dip angles.

By extracting the apparent resistivity values at the depth of the middle position of the target body to calculate Ex, the Ex curves corresponding to each angle were obtained, as shown in Fig. 24.

The Ex curves corresponding to each angle of the high-resistivity target body (Fig. 24 (left)) show that the peak at the left inflection point increases and decreases repeatedly with the increase of the angle in the range of 5°–17°, and increases with the increasing angle greater than 17°. The troughs on both sides of the left inflection point become deeper when the angle increases, with Ex values less than 0, and the trough on the right side is lower than that on the left side. The troughs at the right inflection point also become deeper with the increasing angle, and the peak on the right side is higher than that on the left side, with the corresponding Ex values being less than 0 before 30° on the left peak. For the low-resistivity target body (Fig. 24 (right)), the peaks at the left inflection point increase with the increasing angle, while the troughs at the right inflection point repeatedly increases and decreases with the increasing angle in the range of 5°–17°, and then increases as the angle increases after 17°. The Ex values gradually become greater than 0 towards the right along the topography. This indicates that the geological body accumulates current when the included angle is greater than 180°, leading to an increase in current density, and repels current when the included angle is less than 180°, resulting in a decrease in current density. This topography has a shielding effect on both high-resistivity and low-resistivity target bodies between the left and right inflection points.

The peaks and troughs of the curves represent the relative high and low apparent resistivity. It can be found that the distribution of peaks and troughs is consistent with the distribution of high and low resistivity values. At the position of the target body, the curves do not have corresponding peaks or troughs, but exhibit cross curves with a low-to-high transition under the influence of topography, indicating that this topography has a very strong shielding effect on the target body.

#### 3.2.3 Impact of ridge topography

**Fig. 25:**
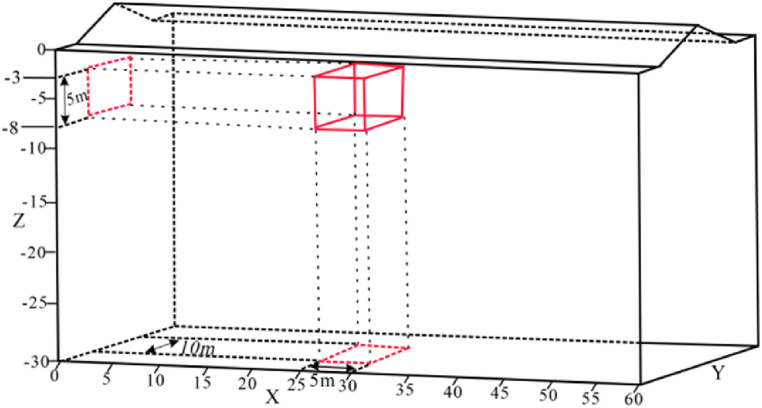
Schematic diagram of the model structure of the ridge model.

This model was used to evaluate the anomalous response characteristics of high-resistivity and low-resistivity anomalies at different steepness levels. The shape and placement of the anomalies are illustrated in Fig. 25. The measuring lines were arranged along the ridge, consistent with the previous setup, and were measured by Wenner array. Measuring lines 3 and 5 were located on the inclined surfaces of the ridge, passing through the edges on both sides of the horizontal projection of the anomalies. Measuring line 4 was positioned at he top of the ridge, passing through the middle of the horizontal projection of the anomalies. Measuring lines 1, 2, 6, and 7 were located on the flat areas on both sides of the ridge, far from the horizontal projection of the target body.

By selecting the apparent resistivity response results from different angles on three selected measuring lines, it can be observed that the apparent resistivity changes significantly with the increase of the angle.

According to Figs. 26a, it is found that the apparent resistivity measured by measuring lines 3 and 5, located on the slopes on both sides, is significantly lower than that by measuring line 4. The high-resistivity anomaly is slightly shifted downwards, but the shape response is well preserved. As the ridge becomes steeper, the anomaly moves downwards, and the apparent resistivity gradually increases. The shape response gradually changes from a regular square to parallel layers as the angle increases. The apparent resistivity of line 4 is highly variable, with the range of high-resistivity anomalies increasing rapidly with the increasing angle, and the shape of the apparent resistivity contour lines gradually changing from an inverted trapezoid to a horizontally oriented ellipse.

**Figs. 26a:**
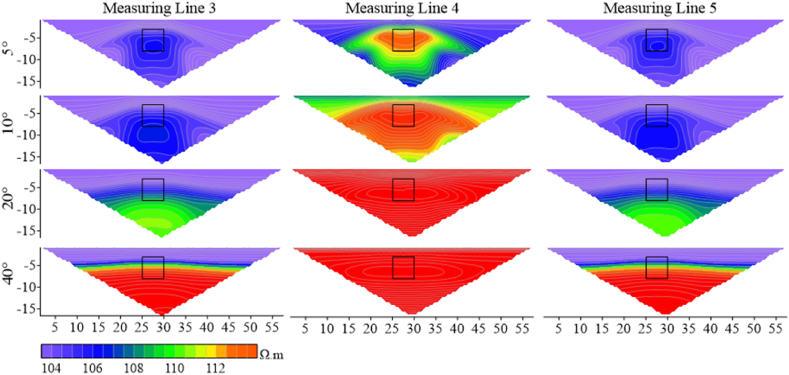
Apparent resistivity response results for high-resistivity target body at various angles for measuring lines 3 **(left)**, 4 **(middle)**, and 5 **(right)**.

Figs. 26b presents the apparent resistivity response results for the low-resistivity target body at different angles on the three measuring lines. As the angle increases, the low-resistivity anomaly gradually decreases until it disappears. At 20°, the low-resistivity anomaly on measuring line 4 disappears, and the whole image turns red representing high resistivity. However, the position of the target body can still be identified on measuring lines 3 and 5 at 20°. This indicates that the apparent resistivity at the ridge position changes dramatically with angles.

**Figs. 26b:**
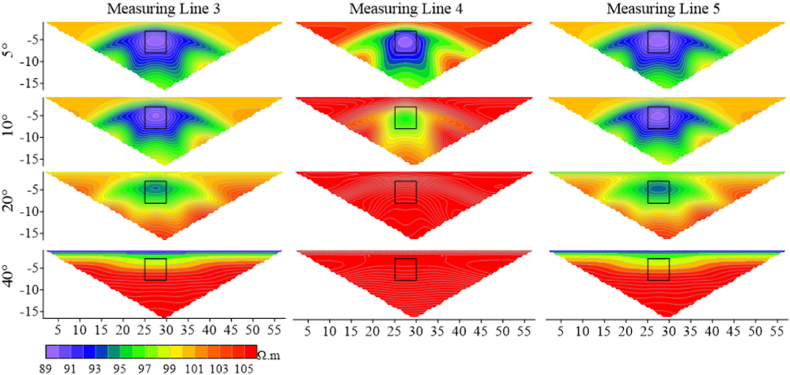
Apparent resistivity response results for low-resistivity target body at various angles for measuring lines 3 **(left)**, 4 **(middle)**, and 5 **(right)**.

To obviously observe the changes in the apparent resistivity of measuring lines 3, 4, and 5 with the terrain, the denominators of the Ex ratio of the three measuring lines were the apparent resistivity of measuring line 4 in flat terrain.

The extracted Ex curves for the three measuring lines are shown in Figs. 26c. The Ex curves corresponding to each angle on the three measuring lines increase with the increase in angle, and troughs appear at the positions where the target bodies are placed, except for 5°. It can be observed that the Ex values for measuring lines 3 and 5 fluctuate repeatedly within the range of less than 20° at the target positions, and then gradually increase after 20°. However, the troughs on measuring line 4 become deeper with the increasing angle, and the trough shapes are relatively flat. This is because the positive terrain similar to a ridge attracts current to pass through the elevated portion, resulting in an increase in current density with the increasing angle. However, the high-resistivity body repels current, leading to a decrease in current density at the target position. Some current is attracted by the ridge topography, thus forming the troughs.

**Figs. 26c:**
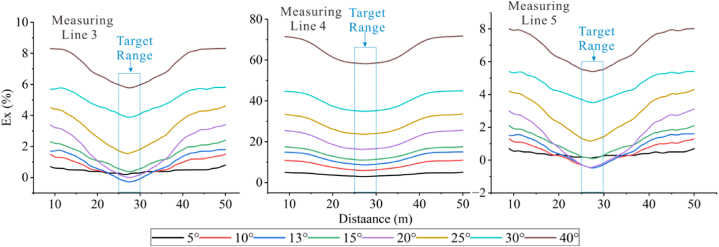
Ex curves corresponding to various angles for the high-resistivity target body on measuring lines 3 **(left)**, 4 **(middle)**, and 5 **(right)**.

In Figs. 26d, it can be seen that the Ex curves corresponding to different angles on the three measuring lines for the low-resistivity target body increase simultaneously with increasing terrain dip angle, and peaks appear at the target positions. The curves of measuring lines 3 and 5 exhibit similar trends from 5° to 10°, and similar trends with increasing peaks from 13° to 15°. They also show similar trends at 25° and 30°, but the peaks at 30° are smaller than that at 25°. There are intersections between 25° and 30° at the side peaks. This indicates that the influence of 30° on the measuring lines located on the slopes is reduced. The 13° and 15° curves for all three measuring lines exhibit similar trends and amplitudes. The reason for this is the same as for the high-resistivity target body.

**Figs. 26d:**
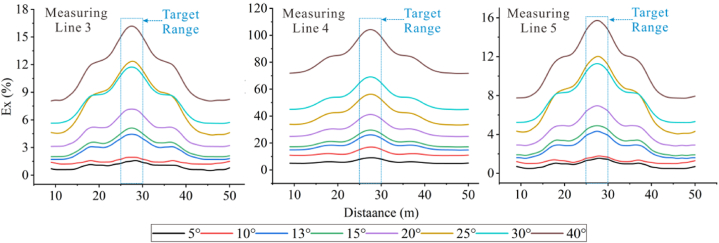
Ex curves corresponding to various angles for the low-resistivity target body on measuring lines 3 **(left)**, 4 **(middle)**, and 5 **(right)**.

It can be seen that the measuring lines 3 and 5 located on the slopes on both sides of the ridge are relatively less affected by the measurement of the low-resistivity target body. Conversely, for the high-resistivity target body, the measuring line 4 on the top of the ridge can identify the location of the high-resistivity anomaly.

#### 3.2.4 Impact of canal topography

Continuing the study of uplifted terrain on apparent resistivity distribution, we next explore the impact of depressed terrain on this distribution to fully understand the influence of terrain variations on the characteristics of subsurface apparent resistivity distribution.

**Fig. 27:**
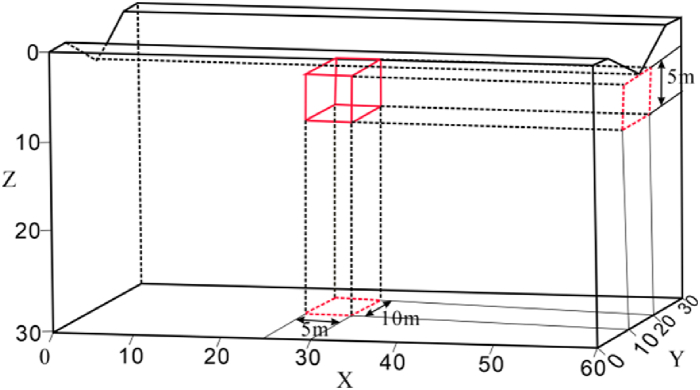
Schematic diagram of the Canal terrain model.

The buried depth of anomalies was −3 m in the model (Fig. 27), and 500 Ω m was set for the high resistivity and 10 Ω m for the low resistivity. The size was the same as the ridge model, located at 25 m–30 m on the X-axis. The Canal terrain bottom had angles of 5°, 10°, 15°, and 20°.

As with the ridge idea, to observe the changes in the apparent resistivity response characteristics of the positions of the three measuring lines with the terrain angles, the denominators of the Ex ratio were set as the apparent resistivity of measuring line 4 on flat ground. Based on the curve changes in the high-resistivity target body (Figs. 28a), it can be seen that the low angle of measuring lines 3 and 5 corresponds to Ex < 0, and as the angle increases Ex increases with it, while the shape of the curve changes from a trough to a peak, with Ex > 0 after 15°. Unlike line 3, line 5 suddenly decreases at 20°, while line 3 increases steadily. The curve shape of line 4 at 5° remains largely unchanged, with only a negative Ex value. As the terrain angle increases, a peak appears at the target position and increases with the increase of the angle. The curves on both sides of the peak decrease as the angle increases. This is because the inflection point at the bottom of the canal repels current, making the density smaller, while the high-resistivity target body repels current, resulting in a larger current density around it. The current flows between the two, leading to an increase in local current density and thus forming the curve shape in Figs. 28a.

**Figs. 28a:**
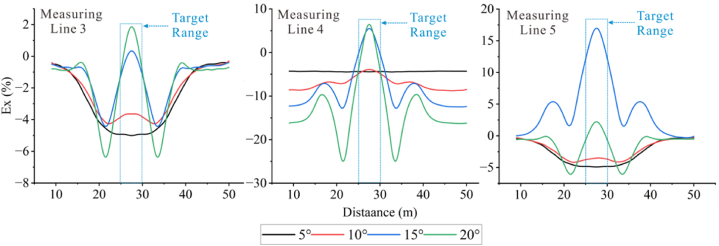
Ex curves corresponding to different depression angles for the high-resistivity target body on measuring lines 3 **(left)**, 4 **(middle)**, and 5 **(right)**.

The curve shape of measuring line 4 in the low-resistivity target body (Figs. 28b) is opposite to that in the high-resistivity target body (Figs. 28a). Due to the decrease in current density at the inflection point of the canal bottom, the low-resistivity target body attracts current, increasing its own current density. Therefore, the trough becomes deeper as the angle increases. Measuring lines 3 and 5, located on the slopes on both sides of the canal, are less affected than the inflection point, so their curve shapes are not completely opposite to those of the high-resistivity target body.

**Figs. 28b:**
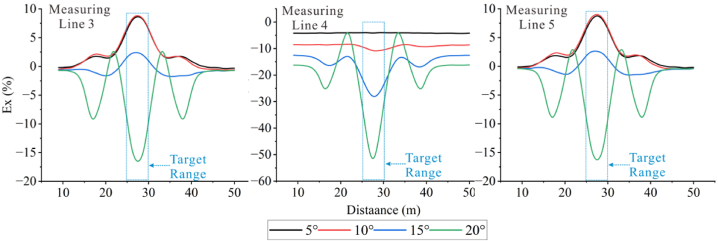
Ex curves corresponding to different depression angles for the low-resistivity target body on measuring lines 3 **(left)**, 4 **(middle)**, and 5 **(right)**.

It is evident that the measurement results at the slope positions are less affected. The response characteristics of the high-resistivity target body are more stable compared to the low resistivity.

#### 3.2.5 Impact of valley terrain

**Fig. 29:**
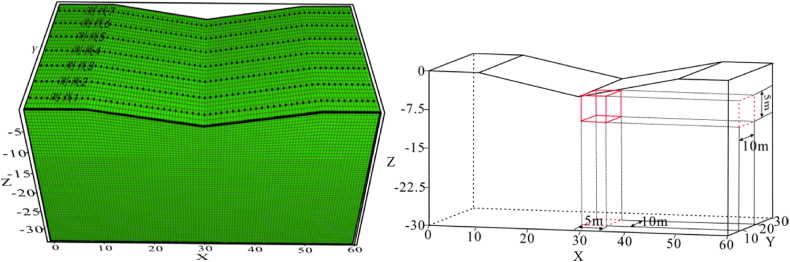
Schematic diagram of the valley model.

Based on the Canal terrain model discussed in Section 3.2.4, we changed the measuring line direction of the Wenner array to make it perpendicular to the terrain strike (see Fig. 29 (left)). The anomaly (see Fig. 29 (right)) has dimensions of 5 m × 10 m × 5 m, with a burial depth of 5 m, and is located below the inflection point of the valley bottom. The resistivity of the anomalies are set as 500 Ω m for the high resistivity and 10 Ω m for the low resistivity, while the background resistivity is 100 Ω m. In addition, the angle variation ranges of the model are 5°, 10°, 15°, and 20°.

**Fig. 30:**
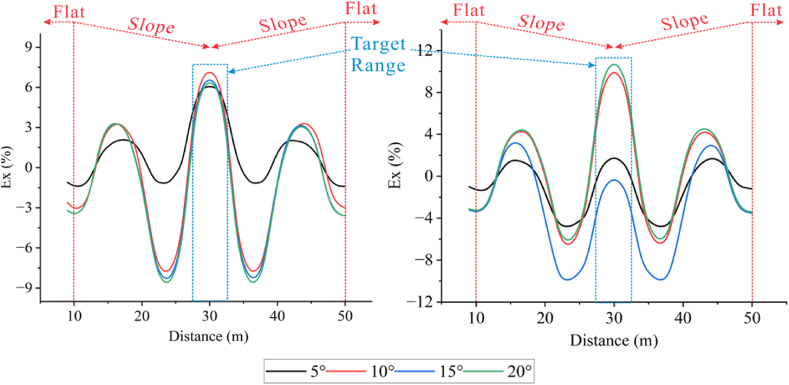
Ex curves corresponding to different depression angles for high-resistivity **(left)** and low-resistivity **(right)** target bodies.

The inflection points are located at X = 10 m, 30 m, and 50 m. The way the Wenner array collects data results in an inverted triangle shape, which reduces the amount of data downward. Here the Ex curve is extracted at a depth of −5.5 m and the X range is between 9 m and 50 m, so the inflection points are reflected at the two ends of the curve. According to Fig. 30, it can be observed that the Ex curves of the high and low resistivity target bodies are similar in shape, with troughs appearing at the inflection points at the junction between the flat and sloping areas (referred to as the left and right inflection points in this section) and peaks occurring at the inflection point at the junction between the two slopes (referred to as the middle inflection point in this section). The curve variations cover the entire X-axis length of the model. The difference is that, based on the Ex of the high resistivity target body in the left Fig., the troughs at the left and right inflection points become deeper as the angle increases, overlapping at 15° and 20°. The middle inflection point, which also coincides with the range of the target body. It can be found that as the depression angle increases, the peaks from 5° to 10° suddenly increase and then decrease, with all Ex values being greater than 0. The troughs at the left and right inflection points of the low-resistivity target body in the right Fig. remain unchanged after the angle increases to 10°. The peaks at the middle inflection points at 5° and 15° correspond to Ex < 0, with Ex > 0 at 10° and 20°, exhibiting a dramatic rise-fall-rise pattern.

A sudden decrease in Ex at 15° occurs for both at the middle inflection points, indicating that there is a critical angle for the depressed terrain, within which the apparent resistivity becomes larger as the angle increases, and beyond which the apparent resistivity decreases with the increasing angle. This is because the increase in Ex greater than 0 enhances the response for high-resistivity anomalies, while the decrease in Ex less than 0 intensifies the response for low-resistivity anomalies.

## 4 Conclusion

Through 3D forward modeling of several models including horizontal level, slope, step, ridge, Canal terrain, and valley, this paper explored the response characteristics caused by the horizontal offset of electrodes and the vertical upward offset induced by terrain. The influencing laws of factors such as the relative position between the measuring lines and the target bodies, the horizontal offset angles, the different terrain dip angles, the resistivity of the targets, and the directions of measuring lines along the terrain were discussed. Through analysis, we summarized the following results:

Impact of horizontal electrode offset. The study reveals that horizontal electrode offset significantly affects the apparent resistivity response, leading to false anomalies. These anomalies become more pronounced as the offset angles and the number of offset electrodes increase. The results show a “gradient variation” pattern in the response, where false anomalies arise due to changes in near-surface current paths. This finding emphasizes the need for cautious electrode placement and recommends multiple measurements with different offsets to improve data reliability.

Impact of topographic relief. The vertical electrode offset caused by terrain leads to distortions and offsets in the apparent resistivity curves and the positions of the anomalies. The study provides a detailed analysis of the effects of different terrain slopes on resistivity data. The results indicate that steep slopes (greater than 15°) require terrain correction or adjustment of measuring line positions to mitigate distortion effects. The study emphasizes the necessity of terrain-specific corrections for accurate subsurface imaging.

Comparative analysis of different electrode configurations. Experimental models by Dipole-Dipole and Wenner arrays show different responses to horizontal and vertical offsets. The study finds that the Dipole-Dipole array is more suitable for detecting low-resistivity anomalies, while the Wenner array performs better for high-resistivity target bodies. Selecting the appropriate array based on the expected underground conditions can effectively improve data quality.

Quantitative assessment and correction demands for offset effects. The study emphasizes the importance of quantitative assessment of offset effects, whose characteristic study serves as the basis for developing correction algorithms. The results of 3D forward modeling provide valuable insights into how different offset conditions affect resistivity data. This information is crucial for optimizing data to interpret techniques and improving the accuracy of geophysical exploration.

While this study has made some findings, there are also some limitations. Firstly, there is a lack of comparison and validation with actual field data. The full paper is based on the analysis of 3D forward modeling data without the analysis of measured data in real geological environments. Only two extreme cases of targets in high and low resistivity were considered, without more complex cases such as middle resistivity values and anisotropies. Real geological conditions are often more complex than modeling. Secondly, only the impacts of irregular terrain on the apparent resistivity response were studied, without involving methods to eliminate these effects. Therefore, our future research will focus on the comparison of actual field data and the optimization of correction algorithms to further explore and improve related research.

## Data availability statement

Data will be made available on request.

## CRediT authorship contribution statement

**Kui Suo:** Writing – review & editing, Resources, Funding acquisition, Data curation. **Mingdong Zhao:** Writing – original draft, Visualization, Data curation. **Menghan Jia:** Resources. **Wenhui Liu:** Resources. **Shizhong Chen:** Software, Formal analysis. **Guizhang Zhao:** Validation.

## Declaration of competing interest

The authors declare that they have no known competing financial interests or personal relationships that could have appeared to influence the work reported in this paper.
